# Benchmarking Generative AI Chatbots in Preventive Medicine: A Comparative Analysis of Accuracy, Completeness, and Inter-Rater Reliability

**DOI:** 10.3390/healthcare14193330

**Published:** 2026-10-06

**Authors:** Nojoud Zoraib Alshahrani, Abdullah Alsabaani, Ali Hassan Almaqsudi, Ali Alshahrani, Naif Moshabub Mosfer Alqahtani, Mazen Abdullah N. Alshahrani

**Affiliations:** 1 Preventive Medicine Residency Program, Saudi Commission for Health Specialties, Aseer Health Cluster, Abha 62512, Saudi Arabia; 2 Department of Family and Community Medicine, College of Medicine, King Khalid University, Abha 61421, Saudi Arabia; aalsabaani@kku.edu.sa; 3 Aseer Health Cluster, Abha 62523, Saudi Arabia; alim@moh.gov.sa; 4 Preventive Medicine Department, Armed Forces Hospital Southern Region, Ministry of Defense Health Services, Khamis Mushait 62413, Saudi Arabia; aalshahrani108@gmail.com; 5 Preventive Medicine Department, Public Health Authority, Abha 62523, Saudi Arabia; naif-454@hotmail.com; 6 Department of Physical Therapy, Saudi German Hospital, Khamis Mushait 62451, Saudi Arabia; aallzyad@outlook.sa

**Keywords:** preventive medicine, large language models, LLM benchmarking, generative artificial intelligence, chatbot evaluation, accuracy, completeness, clinical decision support, inter-rater reliability

## Abstract

**Background/Objectives:** Artificial intelligence (AI) chatbots are increasingly used for healthcare information retrieval, education, and clinical support; however, concerns remain regarding the accuracy and completeness of AI-generated information in preventive medicine, where evidence-based recommendations, epidemiological interpretation, risk assessment, and public health guidance require reliable responses. Comparative evidence across multiple preventive medicine domains remains limited, particularly using standardized expert-validated reference answers. This study aimed to evaluate and compare the accuracy and completeness of responses generated by five AI chatbots across major preventive medicine domains. **Methods:** A comparative cross-sectional benchmarking study evaluated ChatGPT, DeepSeek, Perplexity AI, Claude AI, and Gemini Advanced using 15 expert-validated questions covering General Preventive Medicine, Clinical Preventive Medicine, Epidemiology and Biostatistics, Occupational Medicine, and Social and Behavioral Science. Responses were independently evaluated by two investigators using predefined accuracy and completeness scoring frameworks. Between-model comparisons were conducted using mixed-effects models accounting for clustering by question, and inter-rater reliability was assessed using agreement statistics. **Results:** Claude AI achieved the highest overall accuracy (5.67 ± 0.41) and completeness (2.87 ± 0.35), followed by ChatGPT (accuracy, 5.47 ± 0.66; completeness, 2.67 ± 0.49), whereas Gemini Advanced demonstrated the lowest overall performance. Epidemiology and Biostatistics showed the highest pooled completeness score (2.60; 95% CI: 2.32–2.88). Mixed-effects analysis accounting for clustering by question demonstrated a significant overall difference in accuracy among chatbot models (*p* < 0.001), while inter-rater agreement was high, supporting the consistency of the scoring procedure. **Conclusions:** Substantial variability in accuracy and completeness was observed among the evaluated AI chatbots, with Claude AI and ChatGPT demonstrating comparatively stronger performance within this specific question set. Given the limited number of unique questions and the continuously evolving nature of AI models, these findings should be considered exploratory and should not be generalized as definitive evidence of model superiority or clinical utility. Larger studies using more diverse question sets and standardized evaluation frameworks are required to confirm these findings.

## 1. Introduction

Artificial intelligence (AI) is increasingly integrated into healthcare systems because it can process large volumes of information, support clinical decision-making, and generate human-like responses using advanced natural-language-processing models [1]. Recent developments in generative AI and large language models (LLMs), including ChatGPT, Claude AI, DeepSeek, Perplexity, and Gemini Advanced, have expanded the use of AI chatbots in medical education, patient information retrieval, research assistance, and evidence synthesis [2]. These technologies can generate rapid responses to complex clinical questions and have demonstrated utility across several medical specialties [2]. In preventive medicine, where evidence-based recommendations, epidemiologic interpretation, risk assessment, and public health guidance are essential, the accuracy and completeness of AI-generated information are critical, as incomplete or inaccurate responses may influence healthcare decisions and clinical interpretation [3]. Preventive medicine also emphasizes population-level inference and decision-making under uncertainty [4].

Epidemiological and biostatistical reasoning requires interpretation of disease-frequency and association measures, comparison of risks across populations, and consideration of uncertainty and potential bias [5]. It also requires translating quantitative evidence into screening, prevention, and public health decisions [4]. These tasks differ conceptually from clinical questions that can be addressed primarily through recognition or retrieval of established diagnostic patterns. A valid response must preserve the quantitative and probabilistic relationships underlying the evidence [4]. This distinction is particularly relevant when evaluating LLMs. Fluent and clinically plausible text does not necessarily demonstrate valid statistical inference or probabilistic reasoning, and errors involving numerical relationships or uncertainty may be difficult to recognize within otherwise coherent responses [5]. Epidemiology and biostatistics therefore provide an important domain for assessing whether generative AI systems can address analytically demanding preventive medicine questions rather than merely reproduce familiar medical information [5].

Previous studies have evaluated the performance of AI chatbots across various medical contexts, yielding mixed findings [6,7]. Rao et al. [6] reported that ChatGPT demonstrated substantial utility across the clinical workflow with relatively high diagnostic accuracy, while Marey et al. [7] found high-quality patient education responses generated by ChatGPT for rare cardiovascular diseases. Similarly, Sandmann et al. [8] showed that DeepSeek performed comparably to advanced proprietary language models in clinical decision-making tasks, and Li et al. reported acceptable accuracy and completeness in laboratory medicine support [9]. In radiologic decision-making, Perplexity demonstrated relatively strong performance in pulmonary embolism imaging recommendations according to Woithe et al. [10]; however, Bashah et al. [11] reported lower accuracy for Perplexity in answering salivary-gland-cancer-related questions. Claude AI has also shown favorable performance in complex medical reasoning and in generating concise responses, as demonstrated by Uppalapati et al. [12]. Conversely, Mohamed et al. [13] found that Gemini Advanced-generated responses showed lower alignment with international clinical guidelines than those of other AI systems. These findings collectively indicate considerable heterogeneity in the medical performance of AI chatbots across specialties and clinical tasks [14,15].

Despite growing evidence regarding AI performance in medicine, the reliability of AI-generated responses in preventive medicine remains insufficiently characterized [16]. Preventive medicine encompasses diverse domains, including epidemiology, biostatistics, occupational health, clinical prevention, and behavioral sciences, each requiring accurate interpretation of evidence-based recommendations and a comprehensive contextual understanding [16]. Existing studies have primarily focused on diagnostic reasoning, specialty-specific oncology or radiology questions, and general clinical decision support, rather than on comparative assessment across preventive medicine disciplines using standardized, expert-validated reference frameworks [17,18,19]. Furthermore, relatively limited data are available regarding the completeness of chatbot responses and the reproducibility of scoring methods within preventive medicine contexts [17,18,19]. Addressing these issues is important because variability in response quality may affect the dependability of AI systems used for preventive healthcare education and clinical support.

Therefore, the present study aimed to evaluate and compare the correctness, completeness, and overall response quality of responses generated by ChatGPT, DeepSeek, Perplexity, Claude AI, and Gemini Advanced to expert-validated preventive medicine questions across multiple specialty domains. The study additionally assessed inter-rater reliability and agreement consistency between independent evaluators. It was hypothesized that chatbot models would differ significantly in the accuracy and completeness of their responses and that performance would vary across preventive medicine domains reflecting different knowledge, interpretation, and analytical reasoning demands.

## 2. Materials and Methods

### 2.1. Study Design, Ethics, and Settings

This comparative cross-sectional benchmarking study was conducted between December 2025 and May 2026 at the Department of Preventive Medicine, Ministry of Health, Saudi Arabia. The study evaluated the performance of publicly available generative artificial intelligence chatbots in answering expert-developed preventive medicine questions. Because this study did not involve human participants, patients, biological specimens, identifiable personal data, or patient medical records, institutional policies for non-human-subject research did not require formal institutional ethics committee approval or informed consent. The study investigators and co-authors developed the preventive medicine questions, reference-standard answers, and evaluation framework solely for research purposes. The study was conducted in accordance with accepted principles of responsible research and scientific integrity.

### 2.2. Study Dataset/AI Chatbots and Reference Dataset

The study dataset consisted of responses generated by five publicly available artificial intelligence chatbots to a standardized set of preventive medicine questions. The preventive medicine questions and corresponding reference-standard answers were developed collaboratively by the study investigators, all of whom are co-authors with expertise in preventive medicine. The investigators used a structured consensus process to develop and validate the question set before chatbot evaluation. The responses generated by ChatGPT (GPT-5; OpenAI, San Francisco, CA, USA), Claude (Claude Opus 4.1; Anthropic, San Francisco, CA, USA), DeepSeek (DeepSeek-V3.2; DeepSeek AI, Hangzhou, China), Perplexity AI (Perplexity Pro, model/configuration available at the time of testing; Perplexity AI Inc., San Francisco, CA, USA), and Gemini Advanced (Gemini 3 Pro; Google DeepMind, Mountain View, CA, USA) constituted the analytical dataset. The complete set of 15 expert-validated questions used in this study is provided as Supplementary Material (Table S1), with three questions representing each of the five preventive medicine domains. Publication of the complete question set is intended to facilitate transparent evaluation, independent replication, future comparative benchmarking, and incorporation of the benchmark into subsequent evidence synthesis and meta-analytic research.

### 2.3. Question Development

The investigators developed preventive medicine questions through a structured, expert-driven process to ensure relevance, clinical applicability, and alignment with current evidence-based preventive medicine standards. The study investigators, all investigator-level preventive medicine specialists and co-authors of this manuscript, collaboratively developed English-language questions representing five predefined domains: General Preventive Medicine, Clinical Preventive Medicine, Epidemiology and Biostatistics, Occupational Medicine, and Social and Behavioral Science. The investigator group consisted of investigator-level preventive medicine specialists with more than 10 years of academic and clinical experience in preventive medicine, epidemiology, and public health. Each investigator generated domain-specific questions designed to assess factual accuracy, analytical reasoning, interpretation of preventive medicine principles, and application of evidence-based recommendations. The questions were intentionally structured to reflect clinically and academically relevant preventive medicine scenarios commonly encountered in healthcare practice, public health education, and epidemiological interpretation. To minimize ambiguity and enhance standardization, all questions were formulated using concise, clearly defined terminology consistent with preventive medicine guidelines and educational frameworks described in previous methodological studies evaluating the performance of artificial intelligence in healthcare-related question-answering systems.

### 2.4. Investigator Panel Review

Following initial question generation, all submitted questions underwent review by an investigator panel to assess clarity, domain relevance, clinical significance, and alignment with contemporary preventive medicine recommendations. The investigators did not use artificial intelligence tools during question formulation or reference-answer development to preserve independent expert judgment and minimize incorporation bias. The review process evaluated whether each question adequately represented its assigned preventive medicine domain and whether the expected answer could be objectively verified using recognized evidence-based medical references, preventive medicine textbooks, peer-reviewed literature, and current clinical or public health guidelines. Questions containing ambiguous wording, overlapping concepts, insufficient specificity, or non-standardized terminology were revised or excluded following panel discussion to improve methodological consistency and reproducibility.

### 2.5. Delphi Consensus Process

A modified three-round Delphi consensus method was used to validate the final set of questions and the corresponding reference-standard answers [20]. During the first Delphi round, the study investigators independently reviewed all submitted questions and reference answers for scientific accuracy, clinical appropriateness, and completeness. In the second round, anonymized feedback from all members of the investigator panel was shared electronically, and the investigators re-evaluated the questions after reviewing comments and suggested modifications. The third round finalized consensus and resolved any remaining discrepancies regarding question wording, expected answer content, or domain classification. Consensus was considered achieved when all investigators agreed that the question and reference answer adequately reflected current preventive medicine standards and evidence-based practice recommendations. The Delphi methodology was selected because it is widely recognized as a reliable, structured consensus technique for healthcare research, with expert-derived content validation and methodological standardization.

### 2.6. Reference Answer Development

Reference-standard answers were prepared collaboratively by the study investigators using current evidence-based preventive medicine resources published after the major training cut-off periods of earlier large language models, whenever feasible. Answers were developed using peer-reviewed medical literature, internationally recognized preventive medicine guidelines, epidemiological references, and public health recommendations to ensure scientific validity and consistency. Each reference answer was designed to represent the minimum essential content required for a fully accurate and comprehensive response under the predefined scoring framework. The final reference standards included key preventive medicine concepts, appropriate terminology, and clinically relevant supporting details necessary for objective comparison with AI-generated responses. The investigator panel repeatedly cross-reviewed all reference answers to confirm consistency, factual correctness, and completeness before chatbot evaluation.

### 2.7. Preventive Medicine Domain Classification

The finalized questions were categorized into five preventive medicine domains according to thematic content and specialty focus. General Preventive Medicine questions assessed foundational preventive healthcare concepts and public health principles [21]. Clinical Preventive Medicine questions focused on screening strategies, preventive interventions, and evidence-based clinical prevention practices [22]. Epidemiology and Biostatistics questions evaluated analytical reasoning, interpretation of epidemiological measures, and understanding of statistical concepts relevant to preventive medicine research [23]. Occupational Medicine questions addressed workplace-related health risks, occupational disease prevention, and occupational safety principles [24]. Social and Behavioral Science questions examined behavioral determinants of health, health promotion strategies, and sociobehavioral influences on preventive healthcare outcomes [24]. The investigator panel independently reviewed domain classification to ensure accurate categorization and balanced representation across preventive medicine disciplines.

### 2.8. Validation and Quality Assurance

Quality assurance procedures were implemented throughout the question development and validation process to enhance methodological rigor and reproducibility. During expert validation, preventive medicine questions were also rated for perceived difficulty, benchmark answer quality, and expected completeness using structured expert-assessment scales developed for internal methodological characterization. Difficulty, reference-answer quality, and expected completeness benchmark scores were assessed using 6-point ordinal scales, where higher values indicated greater perceived difficulty, higher reference-answer quality, and greater expected response completeness. Perceived difficulty was evaluated to characterize variation in question complexity within the final question set; however, questions were not prospectively stratified into predefined clinical complexity categories. No separate pilot-testing phase was conducted before formal chatbot evaluation. Instead, methodological refinement was incorporated into the structured investigator review and three-round Delphi process, during which questions were repeatedly assessed for clarity, specificity, domain relevance, scientific accuracy, clinical appropriateness, and expected answer content; ambiguous or insufficiently specific items were revised or excluded before finalization. These measures were used solely to describe the question set and were not included in inferential analyses. These exploratory benchmark indicators were summarized descriptively across preventive medicine domains. All finalized questions and reference answers underwent multiple rounds of expert review to ensure consistency in complexity, clarity, and the expected scope of responses across domains. Standardized formatting and terminology were applied uniformly to all questions prior to submission to the AI chatbots. To reduce procedural variability, each question was entered into the selected AI chatbot platforms using identical prompt instructions requesting the “most accurate and complete answer.” Independent investigators subsequently evaluated chatbot responses using predefined accuracy and completeness scoring criteria developed before data collection. Investigators reviewed scoring discrepancies through discussion and, when necessary, resolved them using the predefined reference standards to maintain consistency and reliability.

### 2.9. AI Chatbot Selection

Five generative artificial intelligence chatbot platforms were selected for comparative evaluation based on their widespread international use, availability of advanced large language model capabilities, and relevance to healthcare information retrieval applications. The selected chatbots included ChatGPT (OpenAI, San Francisco, CA, USA), Claude (Anthropic, San Francisco, CA, USA), DeepSeek (DeepSeek AI, Hangzhou, China), Perplexity AI (Perplexity AI Inc., San Francisco, CA, USA), and Gemini Advanced (Google DeepMind, Mountain View, CA, USA). All platforms were accessed using their most advanced publicly available paid subscription versions during the study period between December 2025 and May 2026. Because large language model versions are continuously updated, the exact model release available at the time of testing was documented internally during data collection. For reproducibility, the contemporaneously recorded model or release identifier, access date, and subscription tier for each platform were retained as part of the study documentation and are provided with the released benchmark materials. Because model availability and server-side configurations may change over time, these identifiers characterize the systems evaluated during the study period and should not be interpreted as representing subsequent platform versions. The selection of these platforms was guided by contemporary market utilization reports and prior healthcare-related AI evaluation studies that identified these systems as among the most frequently used generative AI tools in academic, clinical, and public information settings [25]. All chatbot models were accessed via their officially available web-based interfaces, either through an active subscription or authorized access, to ensure access to their most advanced response-generation capabilities during the study period [25].

### 2.10. Standardization of Response Generation Procedures

To ensure methodological consistency, all chatbot interactions were conducted under standardized testing conditions using identical question wording and prompt instructions across platforms. Each preventive medicine question was entered individually into a newly initiated chat session to minimize contextual carryover effects and memory influence from previous interactions. The use of identical question wording and standardized prompt instructions was intended to maintain comparability across chatbot platforms rather than to characterize the stability of repeated outputs. Chatbot interactions used the default platform settings, with no manual adjustments to temperature parameters or response-generation controls. Testing was conducted through the standard web-based interfaces rather than developer APIs. No investigator-defined temperature, top-p, sampling, or other generation parameters were applied; where these parameters were not exposed to users through the standard interface, their underlying values were platform-controlled and unavailable to the investigators. The activation status of web search, browsing, retrieval, or other tool-use functionality was recorded from contemporaneous testing records for each platform because such functionality may influence generated responses. No additional tools or generation settings were intentionally activated by the investigators beyond those documented for the testing session. When available, memory retention functions were disabled by initiating independent de novo chat sessions for each question. External prompt engineering techniques, follow-up prompts, and iterative clarification requests were not used. This single-turn, zero-shot prompting strategy was intentionally selected to provide a standardized assessment of baseline, out-of-the-box chatbot performance under equivalent input conditions. The design was intended to minimize variability introduced by investigator-specific prompting strategies and did not seek to estimate the maximum performance attainable through optimized human–AI interaction. Accordingly, no role-assigned prompts, structured reasoning instructions, iterative refinement, or other prompt-engineering strategies were applied. Responses were collected from the primary publicly accessible chatbot interface under standard user conditions. The same investigator handled all chatbot entries to reduce inter-operator variability during response-generation procedures. Each chatbot received the standardized instruction: “Provide the most accurate and complete answer,” appended to every preventive medicine question to optimize response comparability and maintain uniform prompting conditions across models. No restrictions were imposed on response length, response style, or formatting structure during chatbot generation procedures. Each of the 15 questions was submitted once to each of the five chatbot platforms, yielding one response for each chatbot-question combination and 75 responses overall. No repeated generations of the same question within a chatbot were performed; therefore, within-model response variability across repeated sessions was not evaluated. This single-generation approach standardized the primary comparison across models but does not characterize the stochastic variability that may occur when identical prompts are repeated. Temperature, top-p, and other sampling parameters were not manually specified because the chatbots were evaluated through their standard public web-based interfaces, which did not expose these parameters for investigator control under the testing conditions used in this study. Accordingly, these parameters remained platform-controlled, and no unverified numerical values were retrospectively assigned. Procedural standardization was maintained by using identical question wording, the same standardized instruction, newly initiated conversations, and no follow-up prompting or investigator-directed modification of response-generation settings across all evaluated platforms.

All responses were generated within the same general time period to minimize potential variability related to major model updates or platform modifications. Questions were administered in English because English-language content constitutes the predominant training corpus for most large language models used in medical applications and allows standardized comparison with evidence-based preventive medicine references. Generated responses were exported directly into a predefined Microsoft Excel database (Microsoft Excel for Microsoft 365, Version 2502, Build 18526.20168; Microsoft Corp., Redmond, WA, USA) without editing, summarization, or linguistic correction to preserve the original chatbot-generated content for subsequent evaluation.

### 2.11. Response Documentation and Data Handling

All chatbot-generated responses were documented verbatim within a structured electronic data collection sheet specifically developed for this study. The database included predefined fields for preventive medicine domain classification, expert reference answers, chatbot-generated responses, investigator-scored outcomes, and adjudication comments, where applicable. Responses were anonymized during evaluation to minimize potential scoring bias from investigators recognizing chatbot identities. Both investigators independently verified the accuracy of data entry before scoring and statistical analyses began. Standardized documentation procedures were maintained throughout the study to ensure reproducibility, traceability, and consistency of chatbot response evaluation across all preventive medicine domains. To support independent inspection and replication of the benchmark, the reproducibility materials include the 15 questions as worded during testing, the corresponding expert-validated reference-standard answers, the standardized prompt instructions, the verbatim, unedited chatbot responses, the predefined scoring rubric, and the response-level scoring dataset. Testing metadata available from the contemporaneous study records, including model or release identifiers, access information, and relevant platform settings, are reported with the benchmark materials; parameters not exposed through the standard web interfaces are identified as platform-controlled and unavailable rather than retrospectively inferred.

### 2.12. Accuracy and Completeness Assessment

Two investigators independently evaluated the accuracy and completeness of chatbot-generated responses using predefined structured scoring frameworks developed before data collection began. All responses were assessed through direct comparison with expert-validated reference-standard answers generated during the Delphi consensus process [26]. Accuracy assessment focused on factual correctness, consistency with preventive medicine guidelines, and overall concordance between chatbot-generated content and the validated reference answers. Completeness assessment evaluated the extent to which the generated responses addressed all essential components of the expected answer and whether sufficient explanatory information was provided to satisfy the predefined benchmark criteria. Responses classified as completely incorrect during the accuracy evaluation were predefined as ineligible for completeness assessment because meaningful completeness scoring was considered inappropriate in the absence of factual correctness. However, all chatbot responses included in the final analysis achieved at least partial accuracy; therefore, completeness assessment was performed for all evaluated responses.

To reduce assessment bias and improve methodological consistency, investigators conducted evaluations independently, using anonymized chatbot responses without disclosing model identity during scoring. Chatbot identity remained concealed from both investigators throughout the independent scoring phase and was not disclosed until independent scoring had been completed. Responses were presented without model-identifying labels during this phase to minimize potential expectancy or brand-related assessment bias. Investigators established standardized scoring instructions and operational definitions before evaluation to ensure uniform interpretation of scoring criteria across preventive medicine domains. When response classification or interpretation was unclear, investigators jointly reviewed the response and referred unresolved discrepancies to the expert investigator panel for adjudication. All scoring procedures used the finalized expert reference standards to ensure consistent evaluation of responses across all chatbot platforms. Before formal scoring, the two evaluators underwent a structured calibration process in which they jointly reviewed the predefined accuracy and completeness rubrics, operational definitions for each score category, expert-validated reference-standard answers, and predefined scoring examples. Any differences in interpretation of the scoring criteria were discussed and resolved before independent evaluation began to establish consistent application of the rubric across preventive medicine domains. Formal scoring was subsequently performed independently using anonymized chatbot responses, with unresolved scoring discrepancies managed through the predefined adjudication procedure.

### 2.13. Scoring System and Outcome Measures

The correctness of chatbot responses was evaluated using a predefined three-point accuracy scale adapted from previously published methodologies for assessing artificial intelligence performance in healthcare information generation [27,28]. Operational scoring examples were predefined before data collection. Responses classified as ‘completely correct’ were required to include all essential evidence-based preventive medicine concepts without factual inaccuracies. ‘Partially correct’ responses contained at least one correct major concept but omitted important information or included minor inaccuracies that did not completely invalidate the response. ‘Completely incorrect’ responses demonstrated major factual errors, misleading recommendations, or failure to address the core question. Potentially misleading information was therefore considered within the overall accuracy classification when it resulted in substantive disagreement with the expert-validated reference standard. However, hallucinations, fabricated or inaccurate citations, outdated guidance, and the clinical severity of potentially unsafe recommendations were not coded as separate prespecified outcomes. Accordingly, the accuracy and completeness framework evaluated response correctness and coverage but was not designed as a dedicated clinical safety or hallucination-assessment instrument. Operationally, a response was classified as completely correct (score: 2) when it contained the essential concepts specified in the expert reference answer without factual error; as partially correct (score: 1) when it identified at least one essential concept but omitted another required component or contained a minor factual inaccuracy that did not invalidate the overall response; and as completely incorrect (score: 0) when major factual errors, misleading recommendations, or failure to address the central question rendered the response inconsistent with the reference standard. Both investigators applied these operational criteria by directly comparing each answer to the same expert-validated reference-standard answer for each question. Accuracy scores for individual questions within each preventive medicine domain were summed to generate domain-level total accuracy scores ranging from 0 to 6. Overall chatbot accuracy across all domains was subsequently calculated from pooled total scores and summarized as the mean and standard deviation. For comparative analyses of overall response quality, question-level accuracy scores were additionally averaged on the original 0–2 scale to facilitate standardized comparisons across chatbot models.

Completeness was evaluated using a structured three-point Likert-type scale applied only to responses achieving an accuracy score of at least 1. Responses were scored as 1 when important aspects of the expected answer were missing or inadequately addressed, 2 when all essential components were addressed with minimally acceptable explanatory detail, and 3 when the response comprehensively addressed all expected components and included additional clinically relevant information beyond the minimum benchmark requirements [28]. Completeness outcomes were summarized at both domain and overall chatbot levels using mean scores and proportions of comprehensive responses. Accuracy and completeness were assessed using predefined ordinal categories intended to distinguish prespecified levels of correctness and response coverage. Because each measure contained only three categories, these scales have limited granularity and may exhibit ceiling effects when responses from high-performing models cluster in the highest category. Accordingly, small qualitative differences among responses assigned the same category cannot be distinguished by these scores, and mean scores are presented primarily as descriptive summaries of the underlying ordinal ratings. Additional outcome measures included Benchmark-Acceptable Response rates, combined performance scores integrating accuracy and completeness metrics, and domain-specific comparative performance analyses between chatbot models. The primary study outcomes were overall chatbot accuracy and completeness scores across all preventive medicine domains. Secondary outcomes included domain-specific performance, Benchmark-Acceptable Response rates, combined performance scores, and inter-rater reliability measures. Benchmark-Acceptable Responses were operationally defined as responses achieving a completely correct accuracy score together with a completeness score classified as at least adequate according to the predefined scoring framework.

### 2.14. Inter-Rater Reliability Assessment

Inter-rater reliability analysis was performed to evaluate consistency and reproducibility between the two independent investigators who scored chatbot-generated responses. Agreement on accuracy and completeness scoring was assessed using quadratically weighted Cohen’s kappa coefficients with corresponding 95% confidence intervals across preventive medicine domains [29]. Intraclass correlation coefficients (ICCs) were also calculated to assess the consistency of continuous scoring outcomes across evaluators and to quantify the overall reproducibility of the scoring methodology. ICC estimates were calculated using a two-way mixed-effects, absolute-agreement model because the two investigators constituted the specific raters used in this study rather than a random sample of raters. Absolute agreement was selected because reproducibility required investigators to assign the same scores, rather than merely demonstrate consistency in the relative ordering of responses. The ICC model and agreement definition are reported explicitly to clarify the inferential scope of the reliability estimates. Discrepant scores identified during the independent evaluation process were reviewed through structured investigator discussion, and unresolved disagreements were adjudicated by consultation with the preventive medicine investigator panel. Reliability analyses were conducted to ensure methodological robustness and to confirm consistency of the predefined scoring framework across all evaluated chatbot responses.

### 2.15. Sample Size

The sample size was calculated a priori using G*Power (version 3.1.9.7; Heinrich Heine University Düsseldorf, Düsseldorf, Germany) based on the originally planned one-way ANOVA comparing five chatbot models. Assuming an effect size of f = 0.40, a two-sided alpha level of 0.05, 80% power, and equal allocation across models, the minimum required sample was 70 response-level observations. The final dataset comprised 75 responses generated from 15 expert-validated questions, with three questions from each of five preventive medicine domains answered by all five chatbot models. Although the final sample exceeded the prespecified minimum, the original calculation was based on response-level observations and did not account for the within-question correlation created by having all models answer the same questions; it is therefore retained as documentation of the original study-planning procedure rather than as evidence of statistical power for the revised mixed-effects analysis. The 75 responses represent only 15 independently sampled questions, and replication across five models does not increase the number of independent content items. Consequently, the study was not independently powered for confirmatory domain-specific comparisons, particularly because each domain contained only three unique questions. The balanced allocation across domains was intended to provide preliminary coverage of major preventive medicine areas rather than comprehensive representation of each specialty. Accordingly, the benchmark should be considered exploratory, and overall and domain-level estimates should be interpreted as comparative performance within the evaluated question set rather than as generalizable measures of model capability across preventive medicine or real-world clinical practice. The revised inferential analysis accounted for question-level clustering, while the limited number of unique questions remained an important constraint on statistical precision and generalizability. Future studies should use substantially larger and more diverse independently sampled, expert-validated question banks to confirm overall and domain-specific performance differences.

### 2.16. Statistical Analysis

All data were entered into a structured Microsoft Excel database and analyzed using SPSS for Windows (version 27.0; IBM Corp., Armonk, NY, USA). Descriptive statistics included means, standard deviations, 95% confidence intervals, frequencies, and percentages, as appropriate, and chatbot performance was summarized at both overall and domain-specific levels. Because each of the 15 questions was answered by all five chatbot models, the 75 response-level observations had a repeated, question-clustered structure and were not treated as independent. Accordingly, inferential comparisons of accuracy, completeness, and combined performance were conducted using mixed-effects models, with chatbot model specified as a fixed effect and question included as a random intercept to account for within-question correlation and between-question heterogeneity; preventive medicine domain was included as a fixed effect where appropriate. Model-estimated differences were reported with 95% confidence intervals and two-sided *p*-values, and multiplicity-adjusted pairwise comparisons were performed following a significant overall model effect. Effect sizes were reported alongside inferential estimates for principal between-model comparisons, where applicable, to characterize the magnitude as well as the statistical significance and precision of observed differences. Statistical significance was defined as a two-sided *p*-value < 0.05.

## 3. Results

Table 1 shows variability in the characteristics and benchmark standards of preventive medicine questions across specialty domains, with Epidemiology and Biostatistics demonstrating the highest difficulty score (4.67 ± 0.58), highest reference answer quality score (6.00 ± 0.00), and highest expert agreement rate of 98.10% (95% CI: 95.40–100.00), indicating greater complexity and stronger consensus compared with other domains (Table 1). Clinical Preventive Medicine also demonstrated relatively high benchmark expectations, with a completeness benchmark score of 4.33 ± 0.58. In contrast, General Preventive Medicine and Social and Behavioral Science showed lower difficulty scores of 2.33 ± 0.58 and 2.67 ± 0.58, respectively, as well as lower reference answer quality scores. Despite these differences, expert agreement remained consistently high across all domains, ranging from 93.40% to 98.10%, supporting the reliability of the expert-derived evaluation framework.

Table 2 shows variation in chatbot accuracy across preventive medicine domains. In the rerun mixed-effects analysis accounting for clustering by question, the overall effect of chatbot model on accuracy was statistically significant (likelihood-ratio χ^2^(4) = 29.22, *p* < 0.001). Using Gemini Advanced as the reference category, ChatGPT had a higher accuracy score (β = 0.467, SE = 0.112, 95% CI: 0.247–0.686, *p* < 0.001), as did DeepSeek (β = 0.267, SE = 0.112, 95% CI: 0.047–0.486, *p* = 0.017) and Claude AI (β = 0.600, SE = 0.112, 95% CI: 0.380–0.820, *p* < 0.001). The difference between Perplexity and Gemini Advanced was not statistically significant (β = 0.133, SE = 0.112, 95% CI: −0.086–0.353, *p* = 0.234). The estimated question-level random-intercept variance was 0.092. Domain-specific mixed-effects analyses did not demonstrate statistically significant overall chatbot-model effects in General Preventive Medicine (*p* = 0.110), Clinical Preventive Medicine (*p* = 0.110), Epidemiology and Biostatistics (*p* = 0.056), Occupational Medicine (*p* = 0.301), or Social and Behavioral Science (*p* = 0.058). These domain-specific findings were interpreted cautiously because each domain contained only three unique questions.

**Figure 1:**
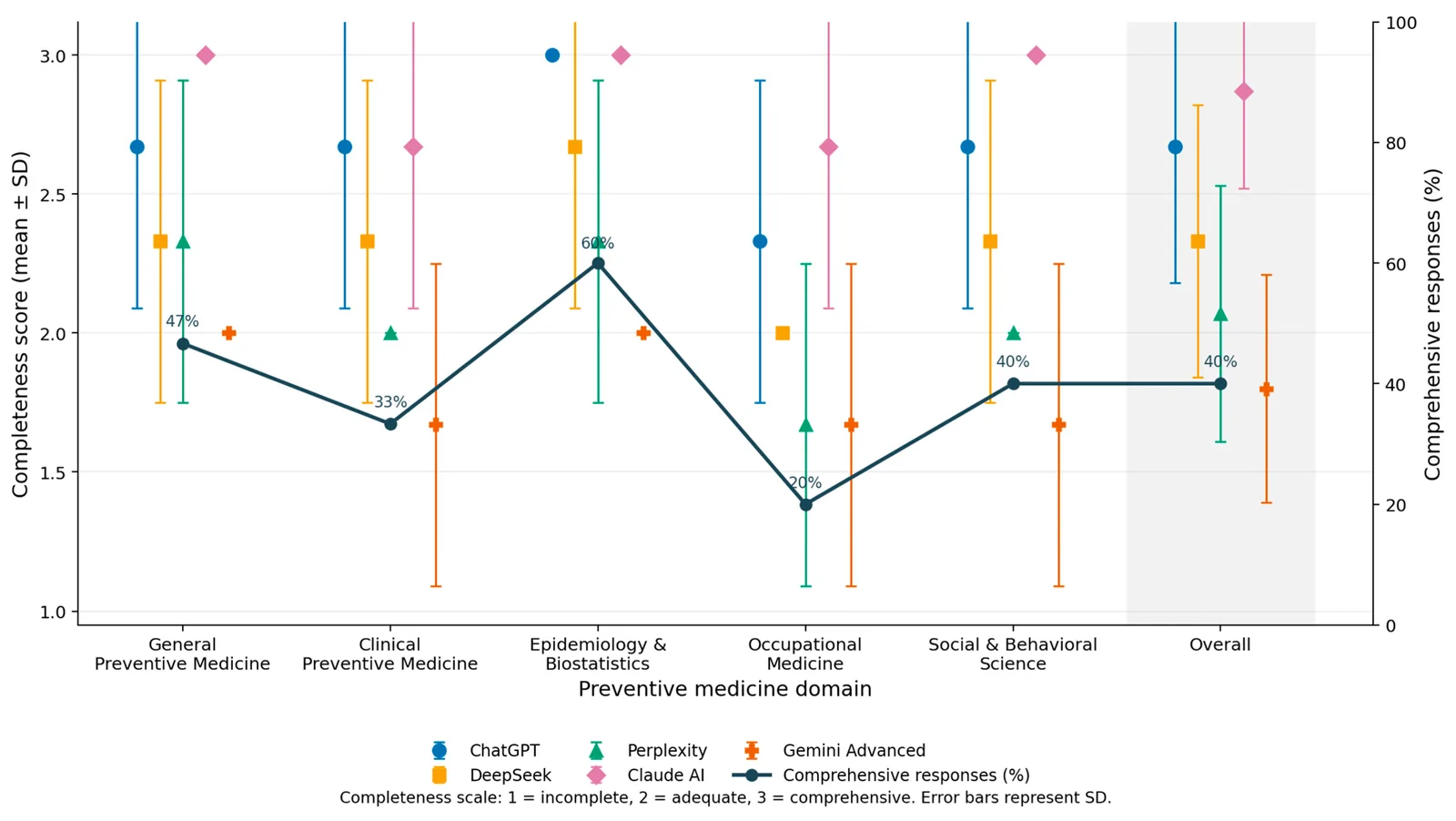
Comparative Completeness Scores and Comprehensive Response Rates of AI Chatbots Across Preventive Medicine Domains.

Results show significant variation in the completeness of AI chatbot responses across preventive medicine domains, with Claude AI consistently demonstrating the highest completeness scores, including an overall score of 2.87 ± 0.35. At the same time, Gemini Advanced had the lowest overall completeness score of 1.80 ± 0.41 (Table 3 and Figure 1). Epidemiology and Biostatistics achieved the highest pooled completeness score of 2.60 (95% CI: 2.32–2.88) and the highest proportion of comprehensive responses (60.00%). Social and Behavioral Science also showed notable descriptive variation between models. Occupational Medicine showed the lowest pooled completeness score of 2.07 (95% CI: 1.74–2.40) and the lowest proportion of comprehensive responses (20.00%). Across all domains, the pooled completeness score was 2.35 (95% CI: 2.21–2.48).

**Figure 2:**
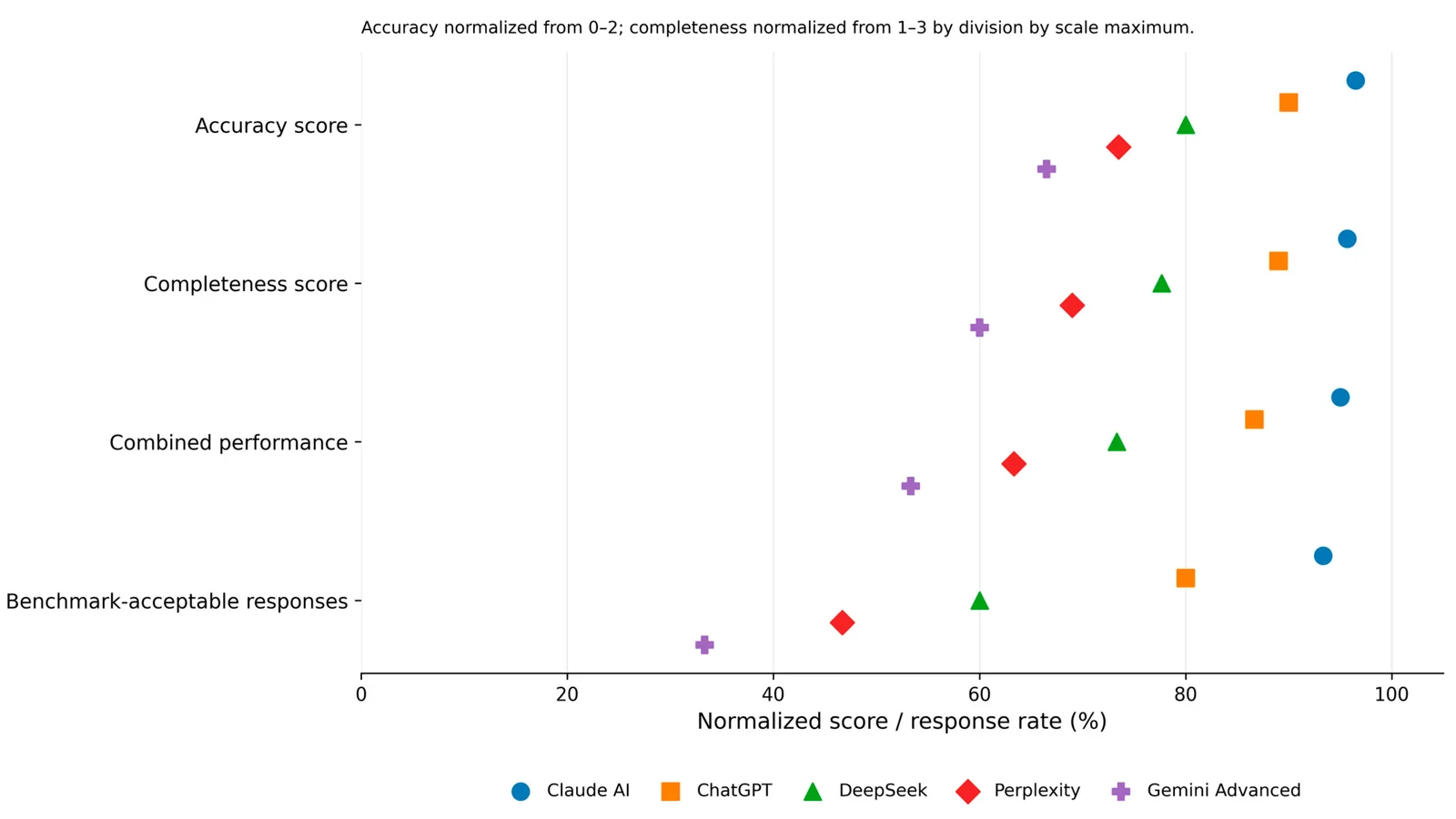
Comparative Benchmark Performance of Artificial Intelligence Chatbots in Answering Expert-Validated Preventive Medicine Questions.

Table 4 demonstrates substantial differences in the overall benchmark performance of the evaluated AI chatbots, with Claude AI ranking first across all evaluated metrics, including the highest accuracy score (1.93 ± 0.26), completeness score (2.87 ± 0.35), combined performance score (95.00 ± 14.02), and Benchmark-Acceptable Response rate (93.33%) (Table 4 and Figure 2). ChatGPT ranked second, achieving an 80.00% Benchmark-Acceptable Response rate and a combined performance score of 86.67 ± 20.85. In contrast, Gemini Advanced showed the weakest performance, with the lowest accuracy score (1.33 ± 0.49), absence of comprehensive responses (0.00%), and the lowest combined performance score of 53.33 ± 18.58. Perplexity and DeepSeek demonstrated intermediate performance, although both produced lower comprehensive response rates compared with Claude AI and ChatGPT. Between-chatbot comparisons revealed statistically significant differences in accuracy, completeness, and combined performance metrics (all *p* < 0.001), highlighting marked variability in the potential informational utility of AI-generated preventive medicine responses.

**Figure 3:**
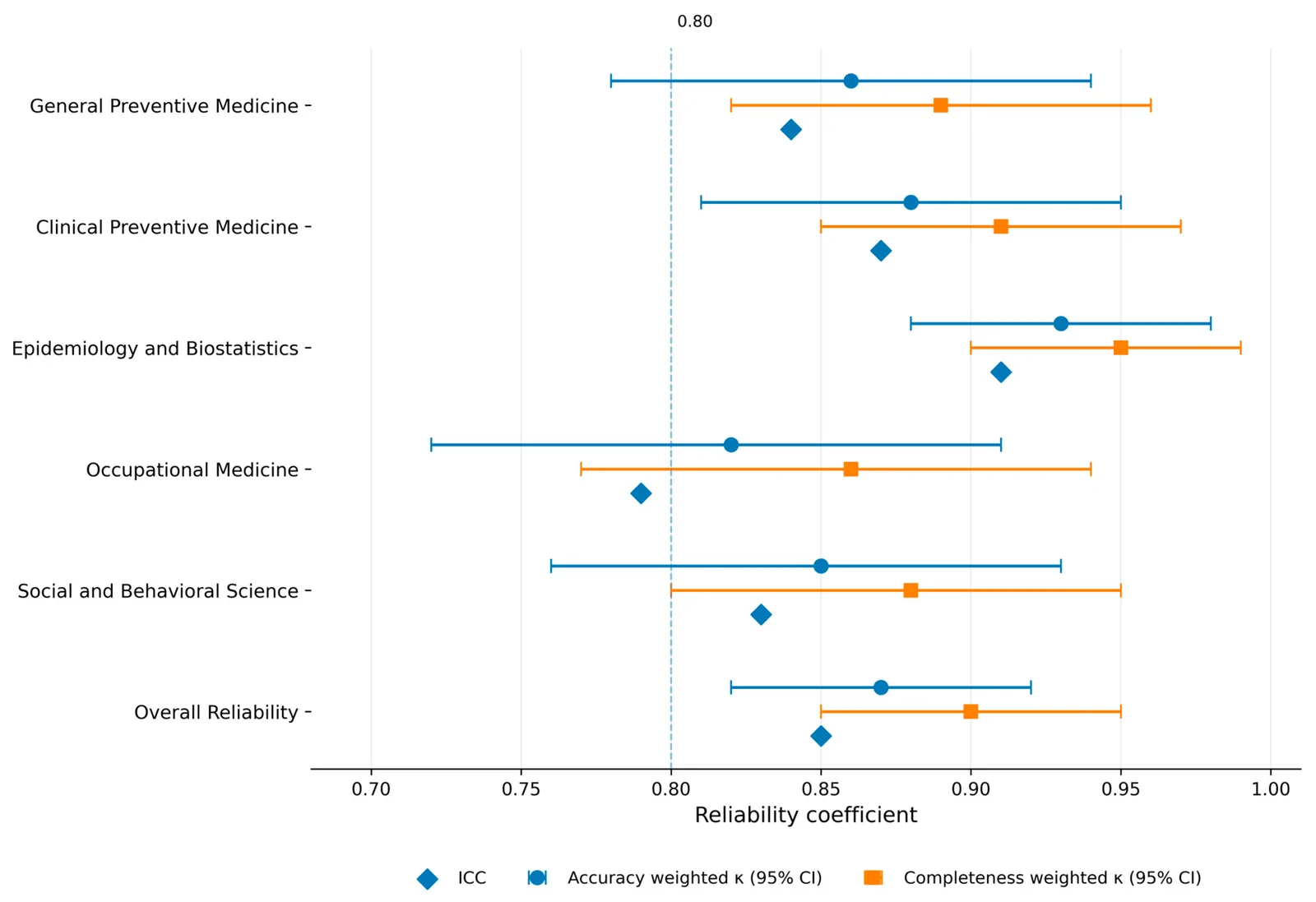
Inter-Rater Reliability of Accuracy and Completeness Scoring Across Preventive Medicine Domains.

Table 5 and Figure 3 demonstrate high inter-rater reliability in the evaluation of AI chatbot responses. Re-analysis using quadratically weighted Cohen’s kappa yielded overall coefficients of 0.876 for accuracy and 0.908 for completeness. Domain-specific weighted kappa coefficients for accuracy and completeness, respectively, were 0.842 and 1.000 for General Preventive Medicine, 0.737 and 0.894 for Clinical Preventive Medicine, 0.865 and 0.894 for Epidemiology and Biostatistics, 0.894 and 0.734 for Occupational Medicine, and 1.000 and 1.000 for Social and Behavioral Science. These results indicate generally strong inter-rater agreement, although the magnitude of agreement varied across domains and scoring dimensions.

## 4. Discussion

This study aimed to evaluate and compare the accuracy, completeness, and overall response quality of responses from multiple artificial intelligence chatbots in preventive medicine across several specialty domains. The findings demonstrated significant variability in chatbot performance, with some models consistently providing more accurate, comprehensive, and Benchmark-Acceptable Responses than others. Claude AI and ChatGPT generally showed superior performance in accuracy and completeness assessments, whereas Gemini Advanced performed comparatively worse across several evaluated domains. Epidemiology and Biostatistics emerged as the domain with the highest overall accuracy, completeness, and expert agreement, while Occupational Medicine showed relatively lower completeness and response quality. Significant differences between chatbot models were observed in both accuracy and completeness outcomes, indicating substantial variability in their ability to provide reliable preventive medicine information. In addition, inter-rater reliability analysis demonstrated excellent agreement and reproducibility between independent evaluators, supporting the consistency and robustness of the applied scoring methodology.

The observed between-model and between-domain variability was consistent with the study’s working hypotheses, although the findings require interpretation in the context of the heterogeneous performance reported for AI chatbots across medical specialties. Previous studies have similarly shown that relative model performance depends on the clinical task evaluated: Claude AI has demonstrated favorable performance in complex medical reasoning, ChatGPT has performed strongly across clinical workflow tasks, DeepSeek has shown performance comparable with advanced proprietary models in some clinical decision-making settings, and the reported performance of Perplexity and Gemini Advanced has varied across specialty-specific evaluations [30,31]. The present findings extend the literature by demonstrating comparable heterogeneity within a standardized, expert-validated preventive medicine benchmark, rather than across unrelated, specialty-specific datasets [31]. Importantly, the comparatively strong performance observed in Epidemiology and Biostatistics, despite its higher expert-rated question difficulty, indicates that perceived task difficulty did not correspond uniformly to poorer chatbot performance in this benchmark. This finding should not be interpreted as evidence that epidemiological or quantitative reasoning is inherently easier for large language models; with only three questions per domain, it instead suggests that performance may depend on the specific content and reasoning demands of the benchmark. Conversely, the comparatively weaker performance in Occupational Medicine indicates that performance in one preventive medicine domain should not be assumed to generalize to others. Overall, these findings support the hypothesis of performance heterogeneity and reinforce the need for domain-specific evaluation before drawing broader conclusions about model capability.

The observed performance differences among chatbot models may reflect differences in their underlying model characteristics and response-generation processes; however, the present benchmarking design cannot determine the mechanisms responsible for these differences. In particular, the study did not examine or verify model training corpora, training procedures, architecture-specific mechanisms, or proprietary optimization strategies. Accordingly, the superior performance observed for Claude AI and ChatGPT in this question set should be interpreted as an empirical difference under the evaluated conditions rather than attributed to specific training data characteristics or model development practices [32]. These models demonstrated greater consistency in generating evidence-aligned preventive medicine responses across multiple specialty domains, particularly in Epidemiology and Biostatistics, where questions required analytical interpretation and structured reasoning [4]. The Epidemiology and Biostatistics domain is particularly relevant to LLM evaluation because correct responses may depend on maintaining probabilistic and quantitative relationships and on interpreting statistical measures in context, rather than solely on retrieving factual medical knowledge [33]. Such tasks can highlight a distinction between linguistic plausibility and valid analytical reasoning, since a response may be coherent yet still misinterpret risk, uncertainty, or an epidemiological measure. Nevertheless, the present findings do not demonstrate an inherent failure of LLMs in probabilistic reasoning. Epidemiology and Biostatistics was rated by the expert panel as the most difficult domain, yet it achieved the highest pooled accuracy and completeness in this benchmark [33]. This finding suggests that some evaluated models performed well on the limited set of analytical questions included in the study, but the small number of questions does not permit conclusions about their general mathematical or probabilistic reasoning capabilities. Future studies should therefore use substantially larger sets of dedicated quantitative problems to evaluate epidemiological calculation, statistical inference, uncertainty interpretation, and robustness to changes in numerical or contextual information.

In contrast, Gemini Advanced and Perplexity showed comparatively lower performance, which may reflect limitations in response precision, contextual stability, or consistency in applying preventive medicine principles to complex questions [34,35]. Similar findings have been reported in previous studies evaluating AI performance in medical decision-making and health information generation. Uppalapati et al. [12] reported that Claude AI generated more concise and clinically coherent responses than competing models in complex medical scenarios, supporting the stronger performance demonstrated in the present findings. Likewise, Rao et al. [6] demonstrated that ChatGPT achieved high diagnostic and clinical workflow accuracy across medical specialties, highlighting its ability to process and synthesize clinical information effectively. Sandmann et al. [8] found that DeepSeek performed comparably to advanced proprietary language models on clinical decision-making tasks, consistent with its intermediate performance in the current analysis. Furthermore, Bashah et al. [11] reported lower accuracy rates for Perplexity in salivary-gland-cancer-related questions compared with other AI models, while Kutbi et al. [36] demonstrated that Gemini Advanced showed weaker alignment with established clinical guidelines in sepsis management, findings that support the comparatively lower accuracy and potential informational utility observed in the present evaluation. Collectively, these results suggest that variability in training strategies, medical knowledge integration, and contextual reasoning capabilities may explain the observed differences in chatbot accuracy and overall clinical performance [1,17].

The differences observed in completeness scores across chatbot models may be explained by variation in each system’s ability to provide comprehensive, structured, and contextually detailed responses rather than brief or narrowly focused answers [37,38]. Claude AI consistently generated the most comprehensive responses, likely due to stronger contextual expansion capabilities and greater ability to integrate supplementary explanatory information relevant to preventive medicine practice [39,40]. ChatGPT also maintained high completeness performance, whereas Gemini Advanced and Perplexity more frequently produced incomplete or minimally detailed responses, particularly in domains requiring broader conceptual discussion, such as Occupational Medicine and Social and Behavioral Science [34]. Another important factor may relate to differences in reinforcement learning and conversational optimization methods that influence how extensively each chatbot elaborates on clinical topics. Similar trends have been documented in previous investigations [34]. Li et al. [9] reported that DeepSeek achieved acceptable completeness in clinical laboratory medicine support, although variability persisted across complex tasks requiring multidimensional interpretation. Uppalapati et al. [12] observed that Claude AI produced more complete and organized answers than other language models in complex healthcare scenarios, supporting the higher completeness scores reported in the present findings. Bashah et al. [11] demonstrated variability in response depth and informational coverage among AI chatbots addressing oncology-related questions, particularly for Perplexity and Gemini Advanced. Additionally, Kutbi et al. [36] found that Gemini Advanced-generated responses were less comprehensive and showed weaker adherence to evidence-based recommendations than ChatGPT responses, which aligns with the lower completeness and clinical acceptability rates observed in the current analysis.

### 4.1. Significance

This study provides comparative benchmarking evidence on the accuracy, completeness, and benchmark-defined response acceptability of widely used artificial intelligence chatbots using a standardized set of expert-validated preventive medicine questions across multiple specialty domains. Performance varied substantially among the evaluated systems under the specified testing conditions, with Claude AI and ChatGPT demonstrating comparatively stronger performance within this question set. These findings may inform future evaluation of AI-generated preventive medicine information and suggest potential value for information retrieval and supplementary educational use; however, they should not be interpreted as evidence of clinical effectiveness, workflow utility, safety, or readiness for deployment. The study did not evaluate real-world clinical encounters, clinician or patient decision-making, patient outcomes, or implementation safety, and the observed differences therefore represent benchmark performance rather than demonstrated clinical utility. This distinction is particularly important in preventive medicine, where inaccurate or incomplete information concerning screening, risk assessment, occupational health, epidemiological interpretation, or population-level recommendations could potentially influence decisions beyond an individual encounter. Accordingly, AI-generated information should not be treated as interchangeable across models or as a substitute for professional judgment, authoritative guidelines, or validated decision-support systems. In healthcare education, chatbots may provide supplementary explanations and support self-directed learning, but incomplete or inaccurate responses could reinforce misconceptions if accepted without verification; their use should therefore be accompanied by critical appraisal and comparison with evidence-based sources. From a system-development and deployment perspective, the observed variation across models and domains highlights the importance of domain-specific validation, transparent reporting of model versions, standardized evaluation, and continued monitoring as AI systems evolve. The high inter-rater agreement observed in this study supports the consistency of the scoring procedure within the present benchmark, rather than the clinical validity of the evaluated systems. Prospective studies using larger and more representative question sets should assess hallucinations, guideline and citation validity, repeated-output stability, clinically consequential errors, and performance in real clinical and public health workflows before conclusions regarding the reliability, safety, or utility of these systems in higher-stakes preventive medicine applications can be established.

### 4.2. Limitations

Several limitations should be considered when interpreting these findings. First, the benchmark comprised only 15 expert-validated questions, with three questions representing each preventive medicine domain. Although this balanced design enabled standardized cross-model comparisons, it cannot capture the full breadth or complexity of preventive medicine, and the study was not independently powered for domain-specific comparisons. Replication of the same questions across chatbots does not increase the number of independently sampled content items; therefore, domain-level estimates and confidence intervals should be considered exploratory and hypothesis-generating rather than definitive evidence of model superiority. Larger studies with substantially more independently sampled questions per domain are needed to provide more precise and adequately powered comparisons. Second, each chatbot generated only one response to each question under the specified testing conditions. The study therefore did not quantify stochastic variation across repeated generations, session-level variability, temporal drift, or longitudinal stability following model updates. Performance may also change because of server-side modifications, geographic deployment differences, and undocumented proprietary adjustments beyond investigator control. Accordingly, individual responses and comparative rankings should be interpreted as observations from the evaluated model versions and testing period rather than invariant estimates of model capability. Future studies should incorporate repeated generations, longitudinal reassessment across model versions and time points, and systematic prompt-perturbation testing. Third, the standardized single-turn, zero-shot prompting strategy provided a controlled baseline comparison but may not reflect real-world professional interactions with generative AI. System-level instructions, role or context specification, few-shot exemplars, structured reasoning prompts, retrieval-supported workflows, follow-up questioning, iterative refinement, and model-specific prompt optimization were not evaluated. Because models may respond differently to contextual scaffolding, identical minimal prompts ensure procedural consistency but not individually optimal performance and may affect both absolute scores and relative rankings. The study therefore cannot determine whether any specific model, including Gemini Advanced or DeepSeek, was disadvantaged by the standardized prompting condition. Future comparisons should evaluate prespecified zero-shot, role-based, few-shot, structured, iterative, and model-optimized prompting strategies to determine the robustness of between-model differences. Fourth, the study evaluated only text-based responses to English-language prompts and did not assess multilingual performance or real-world clinical and public health applications. Although some questions required analytical interpretation and application of preventive medicine principles, clinical reasoning was not measured as a distinct outcome beyond the predefined accuracy and completeness framework. Moreover, hallucinations, fabricated or inaccurate citations, source-attribution errors, outdated guidance, and the clinical severity of unsafe or misleading recommendations were not systematically classified as separate outcomes. Major factual errors or misleading recommendations could reduce accuracy scores, but aggregate accuracy, completeness, and clinical acceptability measures cannot characterize the frequency, type, or potential consequences of these safety-relevant failures. The findings therefore should not be interpreted as demonstrating clinical safety, comprehensive reasoning ability, citation reliability, or deployment readiness. Future benchmarks should incorporate prespecified taxonomies for hallucinations and citation errors, assessment of guideline currency and source validity, and structured grading of potentially harmful recommendations according to clinical severity. Finally, accuracy and completeness were evaluated against expert-derived reference standards. Although these standards were developed through Delphi consensus and the scoring process demonstrated high inter-rater agreement, some degree of expert judgment remained inherent in their development and application. Future research should therefore combine larger and more diverse preventive medicine datasets with standardized evaluation frameworks, multilingual and guideline-updated assessments, repeated testing across model versions and prompting conditions, additional AI systems, and specialty-specific scenarios. Prospective evaluation in clinical, educational, and public health settings will ultimately be required to determine whether benchmark performance translates into reliable and safe real-world utility.

## 5. Conclusions

This comparative study demonstrated meaningful variability in the accuracy, completeness, and overall quality of AI-generated preventive medicine responses across chatbot models and domains. Within the evaluated question set, Claude AI and ChatGPT showed comparatively stronger performance, whereas Gemini Advanced and Perplexity performed less favorably on several measures. Epidemiology and Biostatistics demonstrated the highest overall response quality and expert agreement, while Occupational Medicine showed lower completeness. Significant between-chatbot differences in accuracy and completeness, together with high inter-rater reliability, demonstrated performance heterogeneity while supporting the consistency of the evaluation approach. These findings are exploratory and should be interpreted in the context of the limited number of expert-validated questions and the specific chatbot versions evaluated. Given the rapid evolution of large language models, the observed rankings should not be generalized to future versions or the broader spectrum of preventive medicine practice, and favorable benchmark performance should not be considered evidence of clinical deployment readiness. Because preventive medicine informs screening, risk assessment, epidemiological interpretation, and population-level recommendations, AI-generated information requires validation against authoritative evidence and appropriate professional oversight. Future multicenter studies should use larger and more diverse question sets, repeated evaluations across outputs and model versions, and standardized, transparent benchmarking across prompting conditions and preventive medicine domains. They should also assess clinically consequential errors, including hallucinations, outdated recommendations, and inaccurate source attribution, to determine whether improvements in AI performance translate into reliable support for healthcare education, clinical practice, and public health decision-making.

## Figures and Tables

**Table 1:** Characteristics of Preventive Medicine Questions by Specialty Domain Used for Evaluation of Artificial Intelligence Chatbot Responses in Preventive Medicine.

Specialty Domain	No. of Questions	Difficulty Score (Mean ± SD)	Reference Answer Quality Score (Mean ± SD)	Delphi Consensus Round (Mean ± SD)	Expected Completeness Benchmark Score (Mean ± SD)	Expert Agreement Rate %, (95% CI)
General Preventive Medicine	3	2.33 ± 0.58	4.67 ± 0.58	2.67 ± 0.58	3.33 ± 0.58	95.20 (91.30–98.10)
Clinical Preventive Medicine	3	3.67 ± 0.58	5.67 ± 0.58	2.33 ± 0.58	4.33 ± 0.58	96.80 (93.50–99.10)
Epidemiology and Biostatistics	3	4.67 ± 0.58	6.00 ± 0.00	2.00 ± 0.00	5.00 ± 0.00	98.10 (95.40–100.00)
Occupational Medicine	3	3.33 ± 0.58	5.00 ± 0.00	2.33 ± 0.58	4.00 ± 0.00	94.60 (90.80–97.90)
Social and Behavioral Science	3	2.67 ± 0.58	4.33 ± 0.58	2.67 ± 0.58	3.67 ± 0.58	93.40 (89.20–96.80)

Data are presented as mean ± standard deviation (SD) or percentage with 95% confidence interval (CI). Abbreviations: SD, Standard Deviation; CI, Confidence Interval.

**Table 2:** Mean Accuracy Scores of Artificial Intelligence Chatbots Across Preventive Medicine Domains Based on Expert-Validated Reference Answers.

Preventive Medicine Domain	ChatGPT Mean ± SD	DeepSeek Mean ± SD	Perplexity Mean ± SD	Claude AI Mean ± SD	Gemini Advanced Mean ± SD	Overall Mean Accuracy (95% CI)	Mixed-Effects Model *p*-Value
General Preventive Medicine	5.67 ± 0.58	5.33 ± 0.58	5.00 ± 1.00	5.67 ± 0.58	4.67 ± 0.58	5.27 (4.88–5.66)	0.110
Clinical Preventive Medicine	5.33 ± 0.58	5.00 ± 1.00	4.67 ± 0.58	5.67 ± 0.58	4.33 ± 0.58	5.00 (4.54–5.46)	0.110
Epidemiology and Biostatistics	6.00 ± 0.00	5.67 ± 0.58	5.33 ± 0.58	6.00 ± 0.00	5.00 ± 0.00	5.60 (5.28–5.92)	0.056
Occupational Medicine	5.00 ± 1.00	4.67 ± 0.58	4.33 ± 0.58	5.33 ± 0.58	4.00 ± 1.00	4.67 (4.12–5.22)	0.301
Social and Behavioral Science	5.33 ± 0.58	5.00 ± 0.00	4.67 ± 0.58	5.67 ± 0.58	4.67 ± 0.58	5.07 (4.71–5.43)	0.058
Overall Accuracy Score	5.47 ± 0.66	5.13 ± 0.62	4.80 ± 0.74	5.67 ± 0.41	4.53 ± 0.68	5.12 (4.90–5.34)	<0.001

Data are expressed as mean ± standard deviation (SD) of accuracy scores across preventive medicine domains, using the predefined three-point accuracy scale, aggregated to field totals (range 0–6). Overall mean accuracy values are presented with 95% confidence intervals (CI). Inferential comparisons between chatbot models were performed using mixed-effects models with chatbot model specified as a fixed effect and question included as a random intercept to account for clustering of responses within questions. Abbreviations: SD, Standard Deviation; CI, Confidence Interval.

**Table 3:** Mean Completeness Scores of Artificial Intelligence Chatbots Across Preventive Medicine Domains Using Expert-Validated Reference Standards and a Three-Point Completeness Scale.

Preventive Medicine Domain	Eligible AI Responses, n	ChatGPT Completeness Score, Mean ± SD	DeepSeek Completeness Score, Mean ± SD	Perplexity Completeness Score, Mean ± SD	Claude AI Completeness Score, Mean ± SD	Gemini Advanced Completeness Score, Mean ± SD	Overall Completeness Score, Mean (95% CI)	Comprehensive Responses, %
General Preventive Medicine	15	2.67 ± 0.58	2.33 ± 0.58	2.33 ± 0.58	3.00 ± 0.00	2.00 ± 0.00	2.47 (2.18–2.75)	46.67
Clinical Preventive Medicine	15	2.67 ± 0.58	2.33 ± 0.58	2.00 ± 0.00	2.67 ± 0.58	1.67 ± 0.58	2.27 (1.94–2.60)	33.33
Epidemiology and Biostatistics	15	3.00 ± 0.00	2.67 ± 0.58	2.33 ± 0.58	3.00 ± 0.00	2.00 ± 0.00	2.60 (2.32–2.88)	60.00
Occupational Medicine	15	2.33 ± 0.58	2.00 ± 0.00	1.67 ± 0.58	2.67 ± 0.58	1.67 ± 0.58	2.07 (1.74–2.40)	20.00
Social and Behavioral Science	15	2.67 ± 0.58	2.33 ± 0.58	2.00 ± 0.00	3.00 ± 0.00	1.67 ± 0.58	2.33 (1.99–2.68)	40.00
Overall completeness score across all domains	75	2.67 ± 0.49	2.33 ± 0.49	2.07 ± 0.46	2.87 ± 0.35	1.80 ± 0.41	2.35 (2.21–2.48)	40.00

Completeness was rated on a scale of 1 to 3, where 1 = incomplete, 2 = adequate, and 3 = comprehensive; scores are summarized by specialty domain and chatbot model. Note: Completeness scores were calculated across three questions per domain for each chatbot. The 95% confidence intervals were calculated around the pooled mean completeness scores. Abbreviations: AI; Artificial Intelligence; SD; Standard Deviation; CI; Confidence Interval.

**Table 4:** Comparative Overall Response Quality of Artificial Intelligence Chatbots in Answering Expert-Validated Preventive Medicine Questions Across Accuracy, Completeness, and Combined Potential Informational Utility Metrics.

AI Chatbot	Responses, n	Accuracy Score Mean ± SD	Accuracy Score Mean (95% CI)	Completely Accurate Responses, %	Completeness Score Mean ± SD	Completeness Score Mean (95% CI)	Comprehensive Responses, %	Combined Performance Score, Mean ± SD	Combined Performance Mean (95% CI)	Benchmark-Acceptable Responses, %	Overall Rank
ChatGPT	15	1.80 ± 0.41	1.80 (1.57–2.03)	80.00	2.67 ± 0.49	2.67 (2.40–2.94)	66.67	86.67 ± 20.85	86.67 (75.12–98.21)	80.00	2
DeepSeek	15	1.60 ± 0.51	1.60 (1.32–1.88)	60.00	2.33 ± 0.49	2.33 (2.06–2.60)	33.33	73.33 ± 19.97	73.33 (62.27–84.39)	60.00	3
Perplexity	15	1.47 ± 0.52	1.47 (1.18–1.75)	46.67	2.07 ± 0.46	2.07 (1.81–2.32)	13.33	63.33 ± 18.58	63.33 (53.04–73.62)	46.67	4
Claude AI	15	1.93 ± 0.26	1.93 (1.79–2.08)	93.33	2.87 ± 0.35	2.87 (2.67–3.06)	86.67	95.00 ± 14.02	95.00 (86.67–100.00)	93.33	1
Gemini Advanced	15	1.33 ± 0.49	1.33 (1.06–1.60)	33.33	1.80 ± 0.41	1.80 (1.57–2.03)	0.00	53.33 ± 18.58	53.33 (43.04–63.62)	33.33	5

The combined performance score was calculated by converting accuracy and completeness to 0–100 scales and averaging both components. Confidence intervals for combined performance scores were estimated using a nonparametric percentile bootstrap procedure, thereby maintaining the defined 0–100 bounds of the combined performance scale. Abbreviations: AI; Artificial Intelligence; SD; Standard Deviation; CI; Confidence Interval.

**Table 5:** Inter-Rater Reliability, Agreement Consistency, and Reproducibility of Accuracy and Completeness Scoring Between Independent Investigators Evaluating Artificial Intelligence Chatbot Responses in Preventive Medicine.

Preventive Medicine Domain	Accuracy Scoring Kappa Coefficient	Accuracy Kappa 95% CI	Completeness Scoring Kappa Coefficient	Completeness Kappa 95% CI	Investigator 1 Agreement Rate %, Mean ± SD	Investigator 2 Agreement Rate %, Mean ± SD	Intraclass Correlation Coefficient (ICC)
General Preventive Medicine	0.842	0.78–0.94	1.000	0.82–0.96	94.67 ± 3.25	92.33 ± 4.11	0.84
Clinical Preventive Medicine	0.737	0.81–0.95	0.894	0.85–0.97	95.33 ± 2.89	93.67 ± 3.54	0.87
Epidemiology and Biostatistics	0.865	0.88–0.98	0.894	0.90–0.99	97.33 ± 2.08	95.67 ± 2.73	0.91
Occupational Medicine	0.894	0.72–0.91	0.734	0.77–0.94	92.67 ± 4.52	90.67 ± 5.18	0.79
Social and Behavioral Science	1.000	0.76–0.93	1.000	0.80–0.95	93.33 ± 3.98	91.67 ± 4.36	0.83
Overall Reliability	0.876	0.82–0.92	0.908	0.85–0.95	94.67 ± 3.34	92.80 ± 3.98	0.85

Reliability analysis was performed between two independent investigators using kappa statistics, intraclass correlation coefficients, and agreement rate analysis across all preventive medicine domains. Note: Kappa coefficients were interpreted according to Landis and Koch’s criteria. Agreement rates are expressed as mean percentage agreement ± standard deviation (SD). Intraclass correlation coefficients (ICCs) were used to assess consistency among independent investigators. Paired-samples *t*-tests were used to compare mean differences in agreement between raters across preventive medicine domains. Abbreviations: SD, Standard Deviation; CI, Confidence Interval; ICC, Intraclass Correlation Coefficient.

## Data Availability

The data supporting the findings of this study, including the preventive medicine question set, expert reference-standard answers, scoring rubric, evaluation framework, and study dataset, are openly available in Zenodo at DOI: 10.5281/zenodo.20661125. This DOI represents all versions of the dataset and will resolve to the most recent version.

## Supplementary Materials

The following supporting information can be downloaded at https://www.mdpi.com/article/10.3390/healthcare14193330/s1. Table S1: Expert-Validated Preventive Medicine Question Set.

## Abbreviations

The following abbreviations are used in this manuscript:

AI	Artificial Intelligence
CI	Confidence Interval
Delphi	Delphi Consensus Method
ICC	Intraclass Correlation Coefficient
LLM	Large Language Model
REC	Research Ethics Committee
SD	Standard Deviation
SPSS	Statistical Package for the Social Sciences
