# Supplementary material for: Benchmarking Generative AI Chatbots in Preventive Medicine: A Comparative Analysis of Accuracy, Completeness, and Inter-Rater Reliability
Source: Healthcare (Basel). 2026 Oct 6;14(19):3330. doi: 10.3390/healthcare14193330 (PMC13665411; doi:10.3390/healthcare14193330)
Supplement: Supplementary file 1 [file healthcare-14-03330-s001.zip › healthcare-4410301-supplementary.pdf]

Supplementary Material

Table S1. Expert-Validated Preventive Medicine Question Set

This supplementary table presents a 15-question preventive medicine benchmark comprising three questions from each of five predefined domains.

Question ID	Domain	Question No.	Question text
1	General Preventive Medicine	1	Define herd immunity?
2	General Preventive Medicine	2	How much Folic Acid Supplementation is recommended by US preventive task force for women planning to conceive by US preventive task force to Prevent Neural Tube Defects?
3	General Preventive Medicine	3	For a healthy 50 year old man, which preventive services deliver the greatest long-term benefit?
4	Clinical Preventive Medicine	1	Outline the preventive precautions when responding to hepatitis "A" case?
5	Clinical Preventive Medicine	2	What is Number Needed to Treat (NNT)?
6	Clinical Preventive Medicine	3	Which interventions matter most in 42-year-old male with prediabetes and HbA1c level was 6.0% who wants to prevent progression to diabetes?
7	Epidemiology and Biostatistics	1	A randomized trial reports hazard ratio (HR) 0.72 with a 95% confidence interval between 0.58 and 0.90 for a new intervention. How should this be interpreted?
8	Epidemiology and Biostatistics	2	A clinic has a screening test with 92% sensitivity and 94% specificity for a condition with 2% prevalence. A patient test positive. How likely he is true diseased?
9	Epidemiology and Biostatistics	3	List the key steps of an outbreak investigation.
10	Occupational Medicine	1	Describe the hierarchy of control in occupational health hazard mitigation?
11	Occupational Medicine	2	Describe the clinical findings of noise-induced hearing loss (NIHL)?
12	Occupational Medicine	3	Three employees work at paints factory came to the occupational clinic complaining of cough, wheezing, and shortness of breath. The three worked in the same area of the factory where isocyanates in polyurethane products is used during manufacturing. What is the initial diagnosis?
13	Social and Behavioral Science	1	Identify the theoretical background of the health belief model?
14	Social and Behavioral Science	2	what are the WHO MPOWER measures in short ?
15	Social and Behavioral Science	3	I am 45 years old heavy cigarette smoker for almost 12 years wanting to quit. how would assess my preparedness to start my smoking cessation journey?

Abbreviations: HbA1c, glycated hemoglobin; HR, hazard ratio; NNT, number needed to treat; WHO, World Health Organization.
